# Winter Wisdom

**Published:** 1873-01

**Authors:** 


Winter Wisdom.—Breathe altogether through the nose, with mouth shut, when going out into the cold. The old, the frail and children should have the legs, down to the ankles, and arms, down to the wrists, encased in thick woolen flannel; the space between the shoulder blades behind, where alone the lungs are attached to the body, needs extra protection from the cold more than any other part.

